# Dynamic prediction of early antibiotic treatment failure in ischemic stroke-associated pneumonia: development and internal validation of a 48-h landmark model

**DOI:** 10.1080/07853890.2026.2753847

**Published:** 2026-11-12

**Authors:** Jufeng Qiu, Mingjiao Tang, Sisi Li, Quanchan Li, Cuiling Tang, Xuebin Qin, Haili Feng, Jinsong Li, Tengxiang Zhu, Yuqing Li, Hao Li, Chunhua Lai

**Affiliations:** ^a^ Department of Traditional Chinese Medicine, Affiliated Hospital of Guilin Medical University, Guilin, China; ^b^ Department of Rehabilitation Medicine, Affiliated Hospital of Guilin Medical University, Guilin, China; ^c^ Department of Neurology, Affiliated Hospital of Guilin Medical University, Guilin, China; ^d^ Second Division of Intensive Care Medicine, Affiliated Hospital of Guilin Medical University, Guilin, China

**Keywords:** Antibiotic treatment failure, clinical prediction model, dynamic prediction, ischemic stroke, landmark analysis, stroke-associated pneumonia

## Abstract

**Background:**

Existing stroke-associated pneumonia scores estimate pneumonia occurrence rather than early response after antibiotics have started. We aimed to develop and internally validate a baseline model and a 48-h landmark model for first antibiotic treatment failure between 48 and 72 h after treatment initiation.

**Methods:**

This single-center, retrospective cohort study included consecutive adults with acute ischemic stroke-associated pneumonia treated with empirical systemic antibiotics from December 1, 2021 to December 1, 2025. All included patients were event-free at completion of the 48-h landmark assessment. The primary outcome was first treatment failure by the nominal 72-h reassessment, comprising efficacy-driven antibiotic escalation, persistent clinical instability with evidence of uncontrolled pneumonia, or pneumonia-related death. Ridge logistic regression with a fixed inverse penalty strength (*C* = 1.0) was used, and 500 bootstrap resamples estimated optimism-corrected performance.

**Results:**

Among 199 patients, 102 (51.3%) experienced treatment failure. At baseline, mechanical ventilation before antibiotic initiation and a higher platelet-to-lymphocyte ratio were associated with failure. In the 48-h model, each 20 mg/L increase in C-reactive protein change was associated with greater failure odds (adjusted odds ratio 1.89, 95% confidence interval 1.52–2.34), as were each five-unit increase in neutrophil-to-lymphocyte ratio change (1.98, 1.25–3.13) and escalation of oxygen support (4.56, 1.09–19.01). The optimism-corrected area under the receiver operating characteristic curve increased from 0.697 at baseline to 0.889 at 48 h. Treatment failure was associated with longer hospitalization, higher hospital cost, and longer antibiotic exposure.

**Conclusions:**

In this single-center retrospective study, a structured 48-h reassessment combining routinely available inflammatory trajectories and oxygen-support change improved short-horizon prediction of antibiotic treatment failure. The model should prompt clinical review rather than automatic antibiotic escalation; multicenter external validation and prospective impact evaluation are required before clinical implementation.

## Key Messages

- A 48-h reassessment combining inflammatory trajectories with oxygen-support change markedly improved prediction of early antibiotic treatment failure.
- The optimism-corrected AUC increased from 0.697 at antibiotic initiation to 0.889 at the 48-h landmark.
- The model should trigger structured clinical review rather than automatic antibiotic escalation and requires multicenter external validation.

## 1. Introduction

Stroke-associated pneumonia (SAP) is a common and clinically important complication of acute stroke and is associated with mortality, disability, longer hospitalization, and increased healthcare use [1–4]. Trials of prophylactic antibiotics have not consistently improved functional outcomes, shifting the clinical focus toward timely diagnosis, appropriate empirical treatment, and early reassessment after pneumonia develops [5,6].

Evaluating treatment response is difficult in patients with severe ischemic stroke. Fever, impaired consciousness, aspiration, mechanical ventilation, oxygen requirement, and systemic inflammation may reflect both neurological injury and infection. In hospitalized pneumonia, clinical stability over approximately 72 h is commonly used to judge early response and the need for further diagnostic or therapeutic review [7–9]. However, established stroke-specific tools such as A2DS2, AIS-APS, and PANTHERIS were developed to predict whether pneumonia will occur, not whether initial antibiotic treatment will fail after SAP has been diagnosed [10–14]. The diagnosis of SAP remains challenging because no universally validated diagnostic gold standard is available. The Pneumonia in Stroke Consensus (PISCES) group proposed modified Centers for Disease Control and Prevention (CDC) criteria for SAP occurring within 7 days of stroke onset, incorporating compatible respiratory manifestations, relevant clinical and laboratory findings, and chest imaging features to distinguish probable from definite SAP [2]. The recent European Stroke Organisation guideline further supports standardized diagnostic assessment while acknowledging the remaining diagnostic heterogeneity in this field [1]. In this retrospective study, SAP was identified using compatible respiratory symptoms or signs, inflammatory evidence, and available chest imaging as recorded by the treating team, with consideration of alternative infection sources.

Inflammatory indices derived from routine blood counts may capture complementary aspects of innate immune activation, lymphopenia, and thrombo-inflammatory stress. The neutrophil-to-lymphocyte ratio (NLR), platelet-to-lymphocyte ratio (PLR), systemic immune-inflammation index (SII), and systemic inflammation response index (SIRI) have been associated with SAP and neurological outcomes [15–20]. Because these markers vary with infection burden, stroke severity, and treatment response, their trajectories may be more informative than isolated baseline values.

We hypothesized that a two-stage clinical strategy, consisting of baseline risk assessment followed by structured reassessment at 48 h, would improve prediction of early antibiotic treatment failure. We therefore developed a baseline model and a 48-h landmark model, evaluated the incremental contribution of C-reactive protein (CRP), NLR, and oxygen-support trajectories, and examined the association between treatment failure and hospital resource use.

## 2. Patients and methods

### 2.1. Study design, setting, ethics, and reporting

This retrospective observational cohort study was conducted at the First Affiliated Hospital of Guilin Medical University, Guilin, China. Consecutive eligible admissions between 1 December 2021 and 1 December 2025 were screened. The use of de-identified clinical data was approved by the Ethics Board of the Affiliated Hospital of Guilin Medical University (No. 2026IITLL), which waived informed consent. The study was conducted in accordance with the Declaration of Helsinki. Prospective registration was not applicable because treatment assignment and data collection were not determined by the investigators.

This retrospective observational study used antibiotic initiation as the T0 anchor, with subsequent clinical assessments and laboratory measurements obtained from routine medical records. The operational definitions and analysis dataset were established based on the completed data extraction and record verification. The T72 observation window used in this analysis was 72 ± 8 h after T0, as described below.

The study is reported in accordance with the Strengthening the Reporting of Observational Studies in Epidemiology (STROBE) statement and the Transparent Reporting of a Multivariable Prediction Model for Individual Prognosis or Diagnosis (TRIPOD) principles [21].

### 2.2. Participants

Patients were eligible if they were 18 years or older, had acute ischemic stroke confirmed clinically and by neuroimaging, developed SAP within seven days of stroke onset, received empirical systemic antibiotics, and had sufficient baseline and 72-h outcome information. SAP was operationally identified through independent review of contemporaneous clinical records by two investigators. The assessment considered compatible respiratory symptoms and signs, available chest imaging findings, supporting inflammatory indicators, and exclusion of alternative infection sources, with reference to the diagnostic principles emphasized by the Pneumonia in Stroke Consensus (PISCES) recommendations [2]. Both investigators independently reviewed eligibility records and assessed whether the available diagnostic information was consistent with SAP. Only patients with concordant SAP diagnoses between the two reviewers were included in the final cohort. Separate reviewer-specific diagnostic classifications for the entire screened population were not retained; therefore, Formal inter-reviewer agreement for SAP diagnosis could not be calculated or reported.

Exclusion criteria were lower respiratory tract infection predating the index stroke, another infection source expected to determine early response, antibiotic exposure shorter than 72 h for reasons other than death, indeterminate antibiotic start or outcome status, or a repeated admission. For repeated admissions, only the first eligible admission was retained.

### 2.3. Time origin and landmark framework

T0 was defined as the time of first administration of empirical systemic antibiotics for SAP. In this single-center retrospective study, subsequent blood sampling and clinical observations occurred as part of routine care and could not be scheduled at identical elapsed times. The final data-extraction rules therefore selected the available measurement closest to each target time within T24 = 24 ± 8 h, T48 = 48 ± 8 h and T72 = 72 ± 8 h after T0; where more than one observation was available in a window, the observation closest to the target time was used. As confirmed by the investigators through review of the original charts and antibiotic prescriptions before initial model development, the first efficacy-driven antibiotic changes in the 70 patients fulfilling that component occurred after completion of their T48 assessments. Together with assessment of the remaining endpoint components, this supported entry of all 199 included patients into the T48 landmark risk set. Exact T48 and T72 measurement timestamps were not systematically recorded during data collection, representing a limitation of the present study.

The variability inherent in these ±8-h windows means that the realized interval between T48 and T72 observations was not necessarily 24 h for every patient. Accordingly, the main endpoint was analyzed as a fixed-assessment binary outcome rather than as an exact time-to-event or identical-horizon risk. The (48,72)-h terminology denotes the nominal reassessment framework rather than a verified identical interval for all patients. T24 laboratory measurements were used descriptively, but were excluded from the primary models because of substantial missingness. Future prospective validation will use protocol-scheduled sampling and capture exact draw, clinical-assessment and antibiotic-decision timestamps.

### 2.4. Outcomes

The primary composite endpoint was initial antibiotic-treatment failure after completion of the T48 landmark assessment and by the nominal T72 assessment. Failure required at least one of three nonexclusive components: (1) efficacy-driven escalation, replacement or addition of systemic antibiotics; (2) persistent clinical instability at T72 together with documented uncontrolled pneumonia, ongoing infection activity or poor response; or (3) pneumonia-related death. For each included patient, two investigators independently applied these predefined failure criteria to the relevant clinical records. The two recorded primary-outcome assessments agreed in all 199 patients (100% observed agreement; no disagreement recorded). Agreement on this outcome classification is not an estimate of inter-reviewer agreement for the initial SAP diagnosis. There were no pneumonia-related deaths within the endpoint window. The breakdown and overlap of the endpoint components are reported in Supplementary Table S3. The T72-instability-only sensitivity analysis retained 99 of the 102 composite failures, so it should not be interpreted as an independent endpoint.

Clinical stability at T72 incorporated temperature, heart rate, respiratory rate, systolic blood pressure, oxygenation or oxygen requirement, vasopressor support, mental status, and treating clinicians’ documented assessments. Although T48 oxygen-support escalation preceded the subsequent T72 stability assessment, we acknowledge that these variables are conceptually related because both reflect respiratory deterioration. To evaluate whether this potential predictor–outcome overlap influenced model performance, we repeated both the original composite-outcome and the T72-instability-only analyses after removing the T48 oxygen-support escalation predictor. The full and oxygen-excluded models were compared using the same bootstrap internal validation procedure, including re-estimation of preprocessing parameters and refitting of the ridge regression model within each bootstrap sample (Supplementary Table S4; Supplementary Figure S6). Similar discrimination, calibration, and overall prediction performance were observed after oxygen-predictor omission. We also evaluated T72 persistent clinical instability as an alternative outcome; however, this endpoint retained oxygen-related clinical information and was therefore considered a robustness assessment rather than a completely independent endpoint. Antibiotic escalation decisions were made during routine clinical care and were not guided by the study protocol or prediction model. Treating clinicians were unaware of the study hypothesis, candidate predictor selection, and model development process. However, clinicians were necessarily aware of routinely available clinical information, including laboratory measurements and oxygen requirements, because these data are integral to standard patient management. Therefore, although the predictor information was available as part of usual care, the possibility of residual predictor–outcome overlap and shared clinical information cannot be completely excluded.

### 2.5. Predictors and data processing

Candidate baseline predictors included age, sex, pre-stroke mRS, admission National Institutes of Health Stroke Scale (NIHSS) and Glasgow Coma Scale scores, comorbidities, mechanical ventilation before T0, stroke characteristics, reperfusion treatment, pneumonia timing, diagnosis-to-antibiotic delay, initial antibiotic category, vital signs, oxygen support, and laboratory values. Initial antibiotics were grouped as beta-lactam/beta-lactamase inhibitor combinations, cephalosporins or oxacephems, carbapenems, or fluoroquinolones. Broad-spectrum initial therapy was defined as an antipseudomonal beta-lactam/beta-lactamase inhibitor or carbapenem. Antibiotic comparisons were exploratory because treatment selection was not randomized.

NLR, PLR, monocyte-to-lymphocyte ratio, SII, and SIRI were recalculated only when all required absolute cell counts were available and the lymphocyte count was positive. Changes from T0 to T48 were calculated only when both measurements were observed. Predictor definitions, transformations, and missing-data rules are provided in Supplementary Table S1.

### 2.6. Model development and statistical analysis

Ridge regularization was preferred to Lasso or elastic net because candidate predictors were selected based on clinical relevance, and several clinical and inflammatory variables were potentially correlated. Ridge regression retains clinically relevant predictors while continuously shrinking coefficients rather than automatically setting some coefficients to zero. The principal analysis used a fixed penalty (scikit-learn logistic regression *C* = 1.0; inverse L2-regularization strength) without data-driven tuning [22–24].

As a post hoc penalty-sensitivity analysis, we used fivefold stratified cross-validation separately for the T0 and T48 models, evaluating *C* values of 0.01, 0.1, 1, 10, and 100 with log loss as the selection criterion. This exploratory analysis did not alter the fixed-*C* primary models or their 500-bootstrap internal validation procedure (Supplementary Table S4).

Age, NIHSS, CRP change, and NLR change were rescaled to clinically understandable units. Baseline PLR and diagnosis-to-antibiotic delay were log2-transformed based on statistical considerations, observed right-skewed distributions, and model interpretability. The log2(hours + 1) transformation was used for treatment delay to accommodate zero values. Exact functional forms were not fixed a priori; they were selected during model development according to predictor distributions and modeling requirements. The original untransformed distributions of continuous predictors and formal nonlinear assessment results are provided in Supplementary Table S5. Nonlinear sensitivity checks using cubic B-spline extensions were exploratory and are reported in Supplementary Table S4.

The predictor–outcome sensitivity analyses compared: (i) the primary composite with and without the T48 oxygen-escalation predictor and (ii) T72 persistent clinical instability as an alternative outcome, again with and without the oxygen predictor. Performance comparisons used AUC, Brier score and calibration slope with the same optimism-correction approach as the primary model (Supplementary Table S4; Supplementary Figure S6). Additional exploratory checks examined multiple imputation and a cubic B-spline extension for CRP change.

Discrimination was summarized by AUC, overall prediction error by Brier score, and calibration by smoothed plots and calibration slope [25]. Decision curve analysis assessed net benefit [26]. Internal validation used 500 nonparametric bootstrap samples (seed 20261010): each replicate drew 199 patients with replacement; median imputation and standardization were fitted anew on the resample, followed by refitting the fixed-penalty (*C* = 1.0) ridge logistic model. For each statistic, apparent performance in the bootstrap sample was contrasted with performance of that fitted model in the original sample, and mean optimism was removed from the original apparent result (with direction adjusted for the Brier score) [27]. No penalty tuning was performed within these bootstrap replicates because *C* was fixed in the main analysis. The oxygen-exclusion and alternative-endpoint sensitivity models were validated analogously in 500 bootstrap resamples. A post hoc CRP cubic B-spline model (four quantile knots, fitted within each resample) was examined with the same correction. An exploratory chained-regression multiple-imputation analysis (10 imputations) assessed apparent discrimination only and is not presented as Rubin-pooled inferential validation. Figure 1 displays apparent ROC curves with pointwise 95% percentile bands; Figure 2 displays smoothed apparent calibration curves from the development sample and does not represent optimism-corrected calibration. Optimism-corrected AUC estimates from bootstrap internal validation are reported in Table 1. Conventional multivariable logistic regression provided adjusted ORs and 95% CIs, and log-linear regressions with robust standard errors characterized resource use. The complete ridge regression coefficients, preprocessing parameters, and prediction formula for both the T0 and T48 landmark models are provided in Supplementary Table S2.

**Figure 1.**
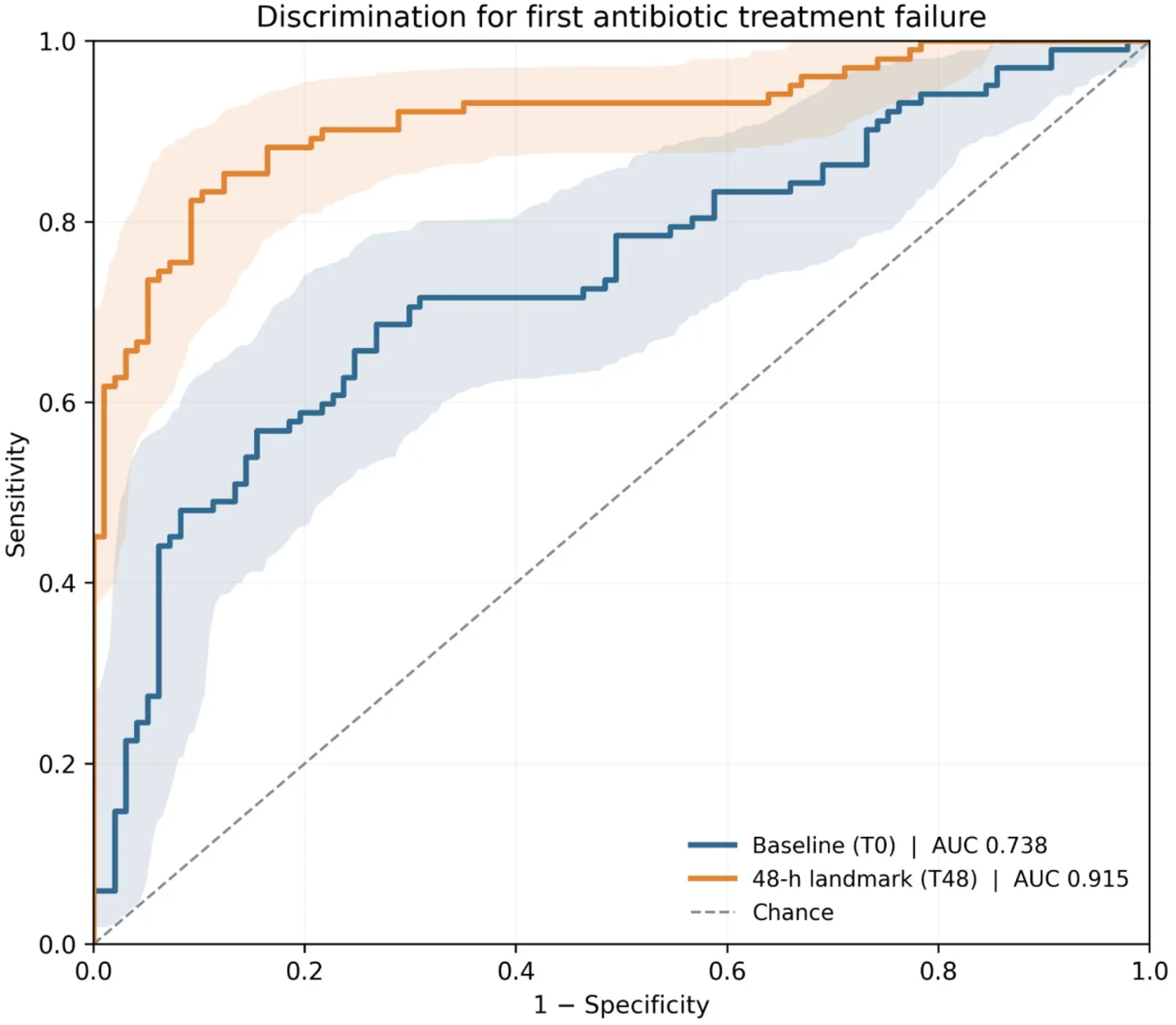
Receiver operating characteristic (ROC) curves for the T0 baseline and T48 landmark ridge models. Solid curves represent apparent discrimination, with 95% pointwise percentile bootstrap intervals (500 resamples).Optimism-corrected AUCs are reported in Table 1.

**Figure 2.**
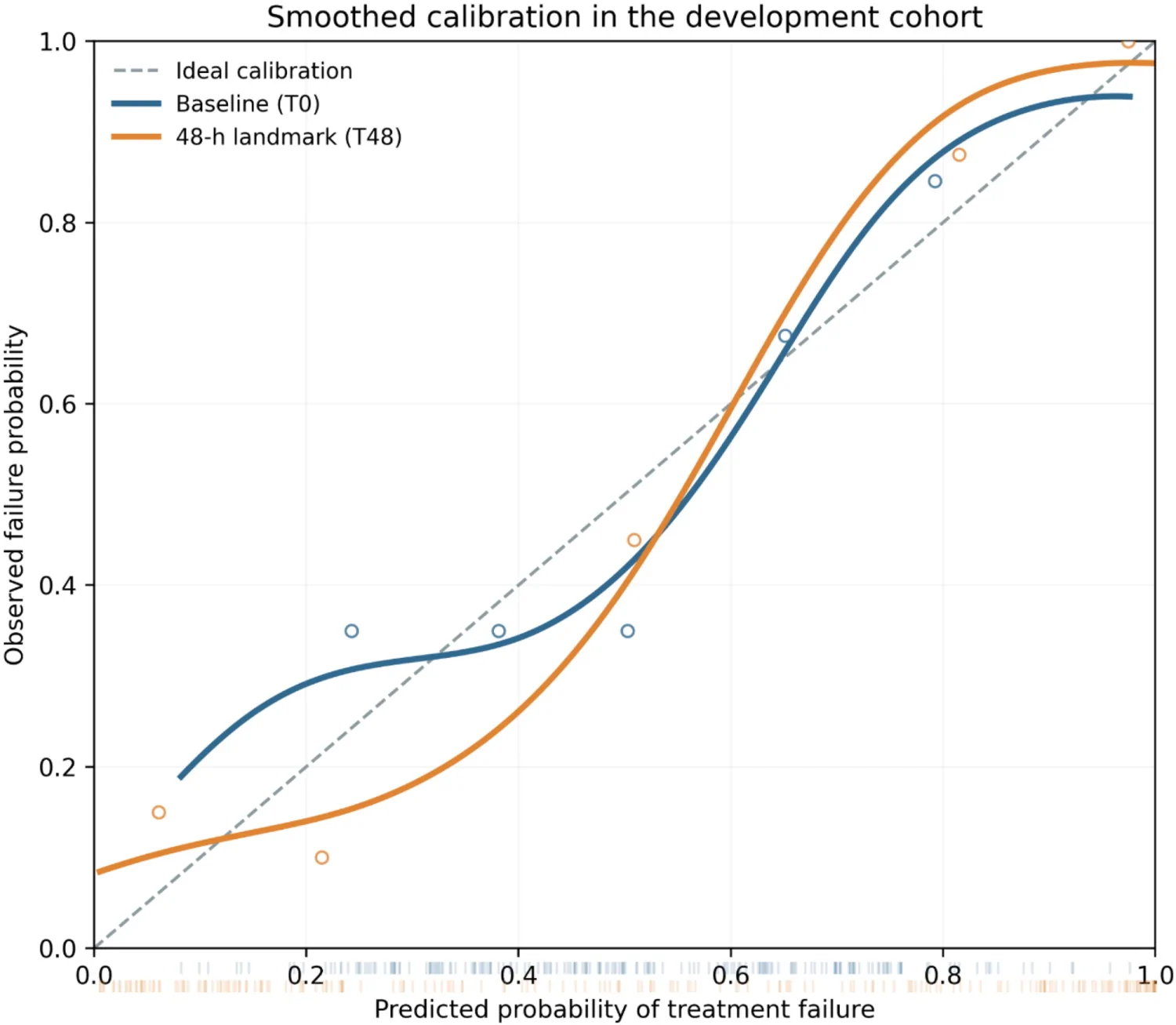
Smoothed calibration curves for predicted first antibiotic treatment failure in the development cohort (n=199). Continuous spline-smoothed lines summarize observed-versus-predicted risk across the model scores; the diagonal denotes ideal calibration.

**Table 1.** Predictive performance after 500-bootstrap internal validation.

Model	Apparent AUC	95% CI lower	95% CI upper	Optimism-corrected AUC	Apparent Brier	Corrected Brier	Corrected calibration slope
T0 model	0.738	0.665	0.807	0.697	0.207	0.227	0.808
T48 landmark model	0.915	0.871	0.953	0.889	0.113	0.133	0.878

Reported AUC estimates are apparent or optimism-corrected as labeled. Model coefficients were estimated with ridge regression and imputation/standardization were repeated inside each bootstrap resample.

## 3. Results

### 3.1. Cohort and baseline characteristics

199 patients were eligible for the T48 landmark (Figure 3). Composite treatment failure occurred in 102 patients (51.3%), while 97 were classified as successes. The nonexclusive components were efficacy-driven antibiotic escalation, replacement or addition in 70 patients, persistent T72 clinical instability in 99, and pneumonia-related death in none; 67 patients met both the antibiotic-adjustment and T72-instability criteria, three met only the antibiotic-adjustment criterion, and 32 met only the T72-instability criterion. Two investigators applied the defined composite outcome criteria, and their separate final outcome records were concordant in all 199 patients. SAP diagnoses were independently reviewed by two investigators, and all included patients had their SAP diagnoses confirmed by both reviewers before cohort inclusion. Formal inter-reviewer agreement across the entire screened population could not be estimated or reported because reviewer-specific diagnostic classifications were not retained during data compilation. Available culture findings are summarized in Supplementary Table S3.

**Figure 3.**
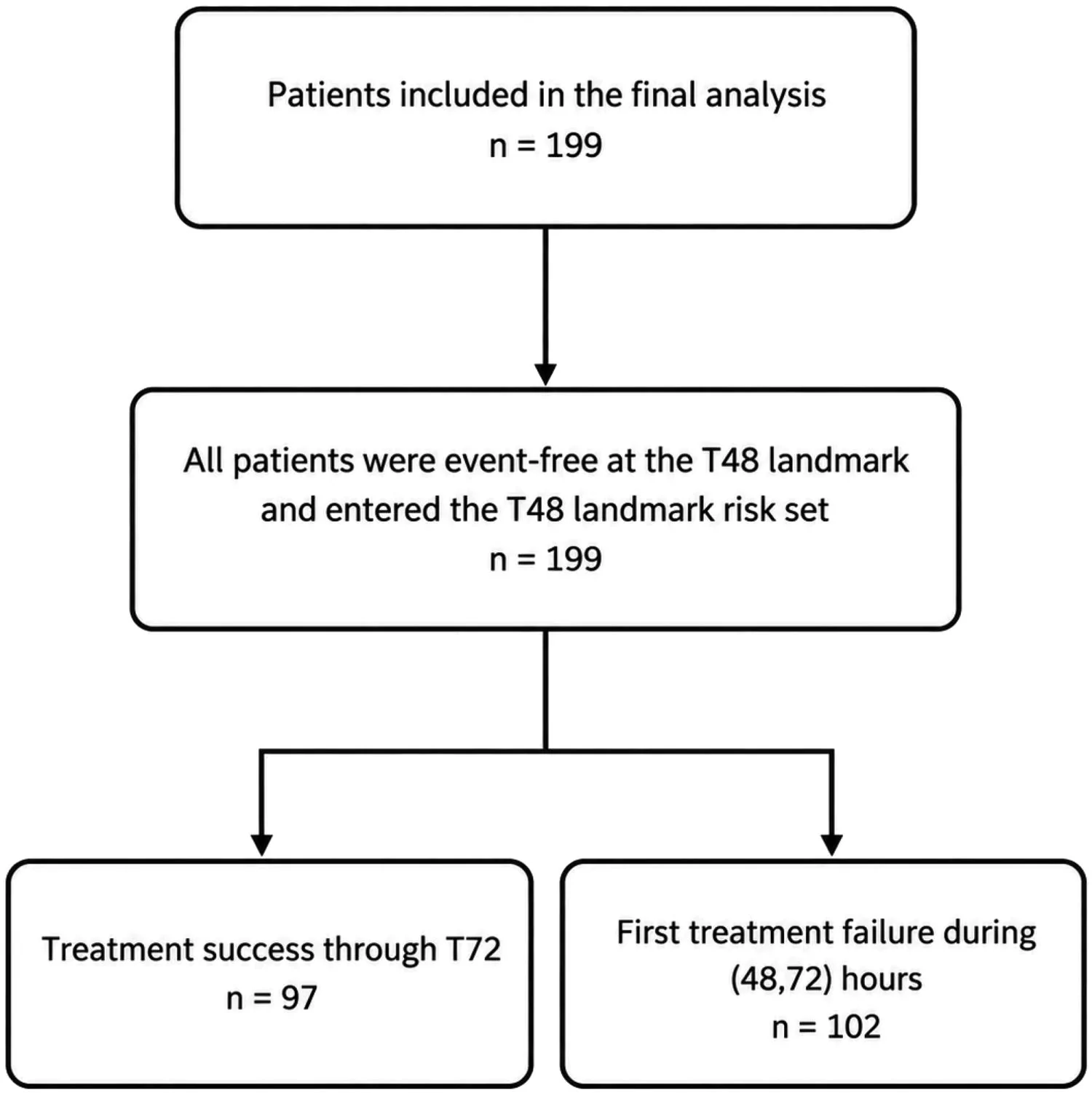
Study flow and construction of the 48-hour landmark cohort.

The median age was 70 years, and 144 patients (72.4%) were men. Compared with patients with treatment success, those with failure had higher pre-stroke mRS scores (*p* = 0.019), higher admission NIHSS scores (*p* = 0.027), lower Glasgow Coma Scale scores (*p* = 0.007), and more frequent mechanical ventilation before T0 (15.7% versus 5.2%; *p* = 0.016) (Table 2).

**Table 2.** Cohort characteristics and dynamic markers by treatment outcome.

Variable	Overall (*n* = 199)	Treatment success (*n* = 97)	Treatment failure (*n* = 102)	*p* value	Available *n*
Age, years	70.00 (63.00–76.50)	70.00 (62.00–77.00)	71.00 (65.00–75.75)	0.304	199
Male sex	144/199 (72.4%)	69/97 (71.1%)	75/102 (73.5%)	0.706	199
Pre-stroke mRS	1.00 (0.00–2.00)	1.00 (0.00–1.00)	1.00 (0.00–2.00)	0.019	198
Admission NIHSS	15.00 (10.25–18.00)	14.50 (9.00–17.00)	16.00 (13.00–19.00)	0.027	198
Admission GCS	12.00 (10.00–13.00)	13.00 (11.00–14.00)	12.00 (10.00–13.00)	0.007	199
Comorbidity count	2.00 (1.00–3.00)	2.00 (1.00–3.00)	2.00 (1.00–3.00)	0.202	199
Mechanical ventilation before T0	21/199 (10.6%)	5/97 (5.2%)	16/102 (15.7%)	0.016	199
Endovascular thrombectomy	91/199 (45.7%)	42/97 (43.3%)	49/102 (48.0%)	0.502	199
Late AISAP (72–168 h)	60/198 (30.3%)	35/97 (36.1%)	25/101 (24.8%)	0.083	198
Diagnosis-to-antibiotic delay, h	0.83 (0.50–10.00)	1.00 (0.50–13.00)	0.67 (0.50–6.75)	0.422	199
Broad-spectrum initial therapy	84/199 (42.2%)	51/97 (52.6%)	33/102 (32.4%)	0.004	199
T0 CRP, mg/L	47.00 (21.00–83.00)	65.00 (27.00–102.00)	35.00 (14.75–72.85)	0.009	195
T0 NLR	9.43 (6.15–14.85)	8.69 (5.87–12.70)	11.60 (6.50–16.18)	0.054	199
T0 PLR	217.23 (153.14–332.64)	200.68 (139.67–297.89)	238.61 (176.98–366.25)	0.021	183
T0 SII	2038.24 (1315.99–3375.98)	1833.22 (1241.08–2838.93)	2332.97 (1435.06–3577.80)	0.041	183
T48 ΔCRP, mg/L	13.00 (−25.18–65.65)	−20.50 (−49.38–0.95)	63.05 (20.25–108.18)	<0.001	192
T48 ΔNLR	−1.74 (−5.24–0.49)	−2.56 (−5.41–-0.78)	−0.08 (−4.74–2.74)	<0.001	199
T48 oxygen escalation	35/199 (17.6%)	10/97 (10.3%)	25/102 (24.5%)	0.009	199

Values are median (interquartile range) or *n*/*N* (%).

AISAP, acute ischemic stroke-associated pneumonia; CRP, C-reactive protein; GCS, Glasgow Coma Scale; mRS, modified Rankin Scale; NIHSS, National Institutes of Health Stroke Scale; NLR, neutrophil-to-lymphocyte ratio; PLR, platelet-to-lymphocyte ratio; SII, systemic immune-inflammation index.

### 3.2. Inflammatory and respiratory trajectories

At T0, PLR was higher in patients with failure than in those with success (median 238.61 versus 200.68; *p* = 0.021), as was SII (2332.97 versus 1833.22; *p* = 0.041). Baseline NLR showed a borderline difference (11.60 versus 8.69; *p* = 0.054).

Dynamic response by T48 was more strongly associated with outcome. Median CRP change was −20.50 mg/L in patients with success and +63.05 mg/L in patients with failure (*p* < 0.001). Median NLR change was −2.56 and −0.08, respectively (*p* < 0.001). Oxygen-support escalation by T48 occurred in 10.3% of successes and 24.5% of failures (*p* = 0.009). Descriptive CRP, NLR, and PLR trajectories are shown in Supplementary Figures S2–S4.

**Figure 4.**
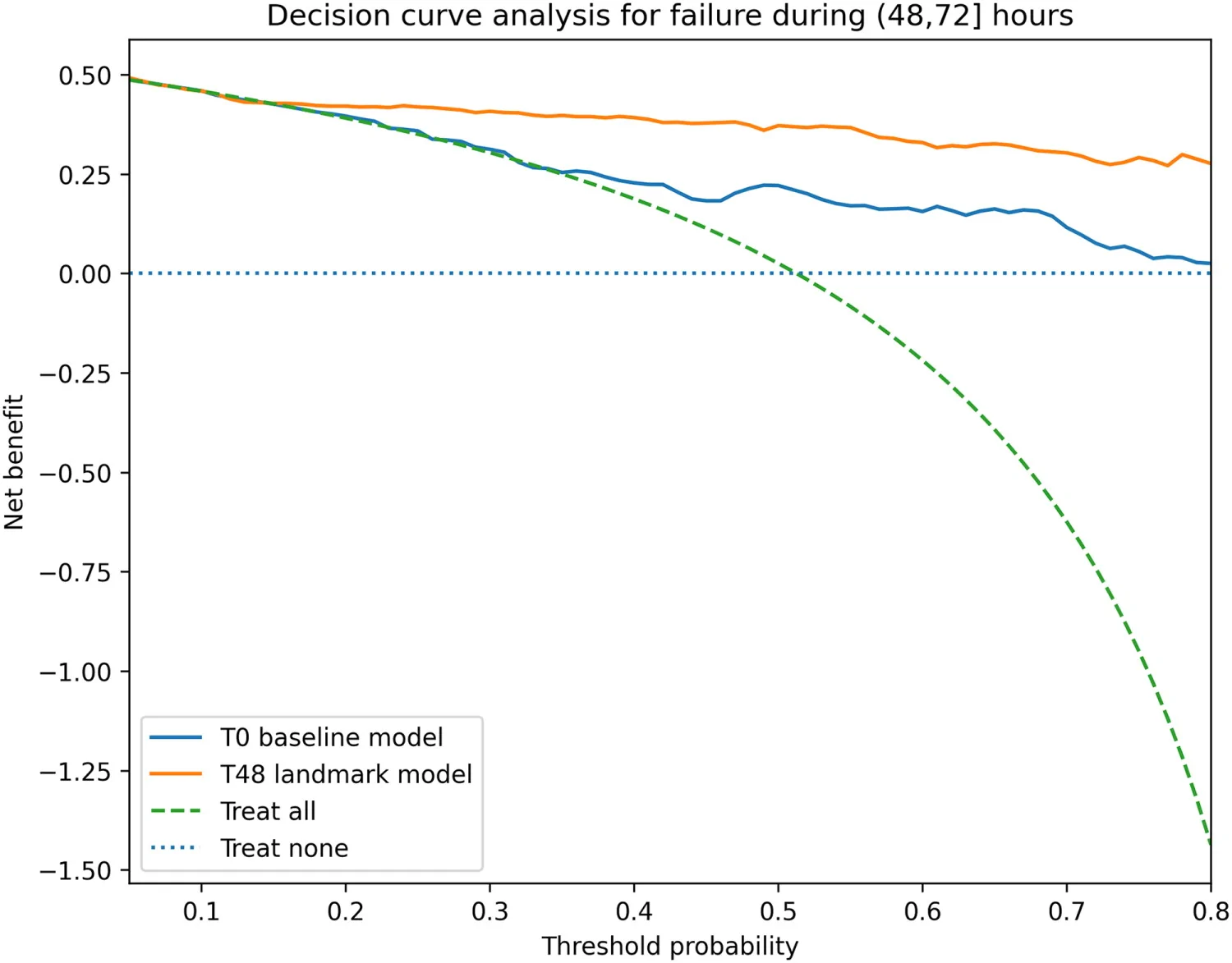
Decision curve analysis comparing the baseline and 48-h landmark models for first treatment failure by nominal T72 assessment. Net benefit is plotted across threshold probabilities; treat-all and treat-none strategies are shown for reference.

### 3.3. Baseline prediction model

In the conventional baseline multivariable model, mechanical ventilation before T0 was associated with higher odds of subsequent failure (adjusted OR 4.65, 95% CI 1.45–14.94; *p* = 0.010), as was baseline PLR per doubling (adjusted OR 1.54, 95% CI 1.03–2.30; *p* = 0.037) (Table 3). Broad-spectrum initial therapy was associated with lower failure odds, but this nonrandomized association is susceptible to treatment-selection and residual confounding.

**Table 3.** Multivariable associations in the baseline and T48 landmark models.

Model and predictor	Adjusted OR	95% CI lower	95% CI upper	*p* value
**Baseline model (T0)**				
T0 model: Age, per 10 years	1.03	0.73	1.43	0.884
T0 model: Pre-stroke mRS, per point	1.49	1.00	2.24	0.052
T0 model: Admission NIHSS, per 5 points	1.29	0.96	1.73	0.092
T0 model: Mechanical ventilation before T0	4.65	1.45	14.94	0.010
T0 model: Late AISAP	0.53	0.26	1.08	0.081
T0 model: Antibiotic delay, per doubling of (*h* + 1)	0.86	0.72	1.03	0.096
T0 model: Broad-spectrum initial therapy	0.30	0.15	0.57	<0.001
T0 model: T0 PLR, per doubling	1.54	1.03	2.30	0.037
**48-h landmark model (T48)**				
T48 landmark model: Age, per 10 years	1.18	0.73	1.93	0.497
T48 landmark model: Pre-stroke mRS, per point	1.22	0.70	2.13	0.475
T48 landmark model: Admission NIHSS, per 5 points	1.09	0.72	1.64	0.678
T48 landmark model: Mechanical ventilation Before T0	3.37	0.88	12.92	0.077
T48 landmark model: Late AISAP	0.54	0.21	1.41	0.207
T48 landmark model: Antibiotic delay, per doubling of (*h* + 1)	0.96	0.76	1.21	0.715
T48 landmark model: Broad-spectrum initial therapy	0.53	0.22	1.29	0.160
T48 landmark model: T0 PLR, per doubling	3.31	1.50	7.32	0.003
T48 landmark model: ΔCRP at 48 h, per 20 mg/L	1.89	1.52	2.34	<0.001
T48 landmark model: ΔNLR at 48 h, per 5 units	1.98	1.25	3.13	0.003
T48 landmark model: Oxygen escalation at 48 h	4.56	1.09	19.01	0.037

The baseline and T48 sections are separated by full-width section labels. Adjusted odds ratios and *p* values derive from conventional multivariable logistic regression, not from the penalized ridge coefficients.

The baseline ridge model had an apparent AUC of 0.738 (95% CI 0.665–0.807) and a re-estimated optimism-corrected AUC of 0.697. The optimism-corrected Brier score was 0.227 and the corrected calibration slope was 0.808 (Table 1).

### 3.4. 48-h landmark model

At the T48 landmark, baseline PLR per doubling remained associated with subsequent failure (adjusted OR 3.31, 95% CI 1.50–7.32; *p* = 0.003). Each 20 mg/L increase in CRP change was associated with 1.89-fold higher odds of failure (95% CI 1.52–2.34; *p* < 0.001), and each five-unit increase in NLR change was associated with 1.98-fold higher odds (95% CI 1.25–3.13; *p* = 0.003). Oxygen-support escalation was also associated with subsequent failure (adjusted OR 4.56, 95% CI 1.09–19.01; *p* = 0.037) (Table 3; Supplementary Figure S1).

The T48 ridge model achieved an apparent AUC of 0.915 and an optimism-corrected AUC of 0.889; the optimism-corrected Brier score was 0.133 and the calibration slope was 0.878 (Table 1). Apparent ROC curves (Figure 1) and smoothed apparent calibration curves (Figure 2) are shown for both the T0 and T48 models; these visualizations represent development-sample performance and are not optimism-corrected. Optimism-corrected AUC estimates derived from bootstrap internal validation are reported in Table 1. Decision curve analysis comparing the baseline and 48-h landmark models is presented in Figure 4.

For CRP-change nonlinearity, exploratory cubic B-spline analyses were performed. The spline model had a corrected AUC of 0.890 and Brier score of 0.129, compared with 0.889 and 0.133 in the original T48 model, while the corrected calibration slope was 0.808 versus 0.878. Given the minimal discrimination improvement and less favorable calibration slope, the original linear ΔCRP specification was retained. The full nonlinear assessment results are presented in Supplementary Tables S4 and S5.

**Table 4.** Patient and health-system outcomes.

Outcome	Treatment success	Treatment failure	Unadjusted *p*
Hospital length of stay, days	12.00 (10.00–15.25)	16.50 (12.00–21.00)	<0.001
Total antibiotic duration, days	7.00 (6.00–8.00)	13.00 (10.25–19.00)	<0.001
Total hospital cost, CNY	44,644 (23,746–113,060)	108,962 (48,082–150,847)	<0.001
Discharge mRS	3.50 (3.00–4.00)	4.00 (4.00–5.00)	<0.001
Adjusted association of failure	Adjusted ratio	95% CI	*p* value
Hospital length of stay	1.33	1.21–1.47	<0.001
Hospital cost	1.50	1.30–1.74	<0.001
Antibiotic duration	1.84	1.64–2.07	<0.001

In the exploratory multiple-imputation analysis with 10 imputations, the T48 model had an apparent AUC of 0.911, compared with 0.915 under the primary median-imputation approach. These in-sample estimates were not optimism-corrected or presented as pooled inferential validation (Supplementary Table S4). In the post hoc cross-validation sensitivity analysis, *C* = 0.1 was selected for T0 and *C* = 1.0 for T48, with corresponding apparent AUCs of 0.739 and 0.915, respectively (Supplementary Table S4).

The sensitivity analyses were performed for robustness assessment only and did not represent external validation. Apparent AUC values from these analyses were not used to replace the optimism-corrected performance estimates of the primary models.

### 3.5. Patient and health-system outcomes

Treatment failure was associated with greater patient and health-system burden. Median hospital stay was 16.5 days in patients with failure and 12.0 days in those with success (*p* < 0.001); median antibiotic duration was 13.0 versus 7.0 days (*p* < 0.001); and median hospital cost was CNY 108,962 versus CNY 44,644 (*p* < 0.001). Discharge disability was also worse in the failure group (Table 4) [28,29].

In adjusted models, failure was associated with 33.1% longer hospitalization (adjusted ratio 1.33, 95% CI 1.21–1.47), 50.2% higher hospital cost (1.50, 1.30–1.74), and 84.2% longer antibiotic exposure (1.84, 1.64–2.07). The distribution of hospital length of stay is shown in Supplementary Figure S5.

## 4. Discussion

This study found that a structured 48-h reassessment substantially improved short-horizon prediction of first antibiotic treatment failure in patients with ischemic stroke-associated pneumonia. The most informative dynamic signals were a rise or lack of improvement in CRP, limited improvement in NLR, and escalation of oxygen support. After internal validation, discrimination increased from 0.697 with baseline information to 0.889 at the 48-h landmark. Treatment failure was also associated with longer hospitalization, greater cost, and substantially longer antibiotic exposure.

The findings address a clinical problem that differs from predicting SAP occurrence. Existing stroke scores identify patients who are likely to develop pneumonia [10–14], whereas clinicians must make a different decision after treatment has begun: whether the patient is improving, requires further investigation, or may need modification of supportive and antimicrobial management. A landmark strategy is appropriate for this purpose because it updates risk using information that becomes available during treatment while preserving the temporal sequence between predictors and the subsequent outcome [30].

Mechanical ventilation before antibiotic initiation identified vulnerability at baseline. In severe stroke, depressed consciousness, impaired airway protection, aspiration, immobility, and organ support can increase both pneumonia severity and difficulty in judging response. Baseline PLR also contributed information, possibly reflecting the combined effects of platelet activation and lymphopenia. However, PLR, NLR, SII, and SIRI share cellular components. Retaining a single baseline inflammatory ratio limited collinearity and kept the model parsimonious.

The dynamic findings are clinically plausible. Rising CRP may reflect persistent inflammation, recurrent aspiration, inadequate source control, resistant organisms, insufficient antimicrobial exposure, or delayed resolution. NLR may capture both persistent innate activation and post-stroke lymphopenia. Oxygen-support escalation adds an organ-level indicator of respiratory deterioration and may be particularly useful when laboratory changes are equivocal. All three measures are routinely available in stroke units and intensive care settings and do not require specialized assays.

The model is not a treatment rule and should not be interpreted as an instruction to automatically broaden antibiotics. Initial broad-spectrum therapy was associated with lower failure odds in exploratory analysis, but treatment was selected by clinicians and was vulnerable to confounding by indication, local prescribing practice, undocumented microbiological risk, and differences in dosing or source control. A high predicted risk should instead prompt structured bedside reassessment, review of microbiological results, reconsideration of aspiration and alternative infection sources, repeat imaging when indicated, and antimicrobial stewardship input.

The composite treatment-failure frequency of 102/199 (51.3%) was higher than rates reported in general hospitalized community-acquired pneumonia studies, including 6% under a narrower clinical-deterioration definition and 14.6% in a non-ICU cohort using antibiotic-change criteria [31,32]. Direct comparisons are limited by differences in study populations, clinical severity, outcome definitions, and ascertainment. The relatively high frequency in our cohort may primarily reflect the broader composite definition, which included persistent clinical instability at T72 and efficacy-driven antibiotic modification rather than mortality alone. Persistent instability accounted for 99/102 (97.1%) composite failures, while antibiotic modification occurred in 70/102 (68.6%); 67 patients met both criteria, and only 3 were classified solely on the basis of antibiotic modification (Supplementary Table S3). Thus, persistent clinical instability was the predominant contributor, while clinician-driven antibiotic changes constituted an additional component of the endpoint. The neurological impairment and respiratory vulnerability of patients with stroke-associated pneumonia may also contribute to difficulty achieving early clinical stability. The overlap between endpoint components explains why the instability-only sensitivity outcome retained 99 events; it does not eliminate potential clinician-dependent ascertainment or predictor–outcome overlap. Available microbiological findings are summarized in Supplementary Table S3. Because microbiological data were incompletely available in this cohort, microbiological information was not incorporated into the analysis of factors contributing to the relatively high treatment-failure rate observed in this study. This represents a limitation of the present study.

The gain in discrimination at T48 is partly expected because inflammatory and respiratory trajectories are measured closer to the subsequent clinical assessment. The similar corrected AUC and Brier scores with and without T48 oxygen escalation indicate that this predictor did not substantially change overall discrimination within the development cohort, although such robustness cannot establish absence of shared clinical information. CRP nonlinearity was borderline in the exploratory test (*p* = 0.050): allowing a cubic-spline term changed corrected AUC minimally and modestly improved Brier score, but the corrected calibration slope was less favorable, supporting retention of the simpler linear form pending external evaluation (Supplementary Tables S4 and S5). In a retrospective study, T0 can be anchored to the first antibiotic administration, whereas routine blood draws and clinical reviews cannot be fixed prospectively. The operational T48 and T72 ± 8-h windows could therefore imply theoretical elapsed intervals as short as 8 h or as long as 40 h, not necessarily an observed range. Exact measurement times were not retained in the analysis dataset, precluding reliable reporting of actual interval distributions or a narrower-window sensitivity analysis. A future prospective study will standardize assessment schedules, retain precise timestamps, and evaluate the model under fixed clinical review intervals.

Limitations include the retrospective single-center setting and internal rather than external validation. Although T0 was defined by the first systemic antibiotic administration, T48 and T72 observations were extracted from routine clinical care within ±8-h target windows; individual sampling and assessment times were not retained in the model dataset. Thus actual prediction horizons may vary and could not be examined in a narrower-window analysis. The 70 efficacy-driven antibiotic changes were verified against source records as occurring after T48 assessment. Antibiotic escalation decisions were made during routine care rather than being influenced by the study model or hypothesis; however, clinicians had access to routine biomarkers and oxygen-support information. Thus, residual shared clinical information and predictor–outcome overlap cannot be completely excluded, particularly because T48 oxygen change and the T72 stability outcome reflect related aspects of respiratory deterioration. The instability-only alternative retained 99/102 original events and is a limited robustness analysis rather than an independent endpoint. Two investigators independently reviewed SAP eligibility before cohort inclusion, and both confirmed SAP in all included patients. Formal inter-reviewer agreement for SAP diagnosis could not be assessed because separate reviewer-specific diagnostic records for the entire screened population were not retained. Treatment-failure classifications were assessed separately and were concordant in all cases. The median-imputation approach does not fully reflect missing-value uncertainty; multiple-imputation and nonlinear CRP analyses are exploratory.

Strengths include the clearly defined T0 anchor, clinically verified timing of first antibiotic changes relative to the T48 landmark, outcome classification using the same explicit failure criteria by two investigators with recorded concordance in 199/199 patients, and a transparent distinction between the original composite and alternative instability outcome. Predictors, missing-value preprocessing and standardization were re-estimated during bootstrap model validation. The penalized-model specification is provided to facilitate external validation.

For clinical practice, the findings support reassessment at 48 h rather than reliance on a single baseline inflammatory value. A combination of biomarker trajectory and respiratory-support change may help prioritize patients who need closer review. Before implementation, however, the model requires multicenter external validation, local recalibration, and prospective evaluation of whether its use improves patient care without promoting unnecessary antibiotic escalation.

## 5. Conclusions

In ischemic stroke-associated pneumonia, changes in CRP and NLR together with oxygen-support escalation at 48 h markedly improved prediction of first antibiotic treatment failure by the subsequent nominal 72-h assessment. This pragmatic two-stage approach may support targeted clinical reassessment, but it requires multicenter external validation and prospective impact evaluation before routine use.

## Supplementary Material

Supplemental Material

Figure S5.tiff

table S5.docx

table S3.docx

Figure S6.tiff

Figure S3.tiff

table S2.docx

table S1.docx

Figure S4.tiff

STROBE_Checklist.docx

Figure S2.tiff

Figure S1.tiff

table S4.docx

## Data Availability

The data supporting the findings of this study are available from the corresponding author upon reasonable request.
