# Supplementary material for: Dynamic prediction of early antibiotic treatment failure in ischemic stroke-associated pneumonia: development and internal validation of a 48-h landmark model
Source: Ann Med. 2026 Nov 12;58(1):2753847. doi: 10.1080/07853890.2026.2753847 (PMC13761045; doi:10.1080/07853890.2026.2753847)
Supplement: Supplementary file 1 [file IANN_A_2753847_SM5991.docx]

Table S4. Post hoc model sensitivity analyses of endpoint overlap, missing values, CRP functional form, and ridge penalty choice

| **Analysis / endpoint** | **n / events** | **Apparent AUC; Brier; slope** | **Corrected AUC; Brier; slope** | **Interpretation** |
| --- | --- | --- | --- | --- |
| Primary T0 (baseline) | 199 / 102 | 0.738; 0.207; — | 0.697; 0.227; 0.808 | Original main Table 3 bootstrap results |
| Primary T48 (full) | 199 / 102 | 0.915; 0.113; 1.139 | 0.889; 0.133; 0.878 | 500-bootstrap aligned sensitivity-pipeline comparator |
| Composite outcome: omit T48 oxygen escalation | 199 / 102 | 0.915; 0.115; 1.134 | 0.892; 0.133; 0.914 | AUC ranking changed minimally after removing predictor |
| T72 instability only: full T48 model | 199 / 99 | 0.930; 0.103; 1.170 | 0.907; 0.122; 0.881 | 99/102 cases overlap with original composite |
| T72 instability only: omit T48 oxygen escalation | 199 / 99 | 0.927; 0.104; 1.163 | 0.906; 0.122; 0.925 | More directly addresses both overlap concerns, not definitive |
| CRP change modeled with cubic B-spline (4 quantile knots) | 199 / 102 | 0.919; 0.106; 1.078 | 0.890; 0.129; 0.808 | 500-bootstrap exploratory; knots re-estimated |
| Multiple-imputation sensitivity, 10 imputations | 199 / 102 | 0.911; —; — | Not estimated | Exploratory apparent AUC; not Rubin-pooled validated performance |
| Penalty sensitivity: T0 (post hoc five-fold stratified CV) | 199 / 102 | C=0.1; AUC=0.739 | Not estimated | Log-loss selection; apparent AUC only |
| Penalty sensitivity: T48 (post hoc five-fold stratified CV) | 199 / 102 | C=1.0; AUC=0.915 | Not estimated | Log-loss selection; apparent AUC only |
| CRP-change nonlinearity LRT | 199 / 102 | P=0.050 | Not applicable | Exploratory unpenalized GLM test |
| NLR-change nonlinearity LRT | 199 / 102 | P=0.616 | Not applicable | Exploratory unpenalized GLM test |

The primary ridge models used a fixed inverse L2 regularization parameter (C = 1.0), without penalty tuning during model fitting or the 500-bootstrap internal validation. The post hoc five-fold stratified cross-validation sensitivity analysis compared C = 0.01, 0.1, 1, 10, and 100 using log loss for selection; it selected C = 0.1 for T0 and C = 1.0 for T48. AUCs reported in the penalty-sensitivity rows are apparent (in-sample) values, not cross-validated or optimism-corrected estimates.
