# Supplementary material for: Dynamic prediction of early antibiotic treatment failure in ischemic stroke-associated pneumonia: development and internal validation of a 48-h landmark model
Source: Ann Med. 2026 Nov 12;58(1):2753847. doi: 10.1080/07853890.2026.2753847 (PMC13761045; doi:10.1080/07853890.2026.2753847)
Supplement: Supplementary file 2 [file IANN_A_2753847_SM5994.docx]

**STROBE Statement - Checklist for Cohort Studies**

| **Item** | **No.** | **Recommendation** | **Location in manuscript** |
| --- | --- | --- | --- |
| Title and abstract | 1a | Title and abstract | Study design stated in the title and Abstract Methods. |
| Title and abstract | 1b | Title and abstract | Structured abstract provides balanced methods, effect estimates, model performance, and conclusions. |
| Background/rationale | 2 | Background/rationale | Introduction, paragraphs 1-3. |
| Objectives | 3 | Objectives | Introduction, final paragraph. |
| Study design | 4 | Study design | Patients and Methods, section 2.1. |
| Setting | 5 | Setting | Patients and Methods, sections 2.1 and 2.3; exact calendar dates remain to be inserted. |
| Participants | 6a | Participants | Patients and Methods, section 2.2. |
| Matched studies | 6b | Matched studies | Not applicable; no matching. |
| Variables | 7 | Variables | Patients and Methods, sections 2.4-2.5. |
| Data sources/measurement | 8 | Data sources/measurement | Patients and Methods, sections 2.3 and 2.5. |
| Bias | 9 | Bias | Sections 2.5-2.7 and Discussion limitations. |
| Study size | 10 | Study size | Section 2.6: all eligible patients included; no prospective calculation; model parsimony based on event count. |
| Quantitative variables | 11 | Quantitative variables | Sections 2.5-2.7. |
| Statistical methods | 12a | Statistical methods | Sections 2.6-2.7. |
| Subgroups/interactions | 12b | Subgroups/interactions | Exploratory antibiotic comparisons described in Methods and Supplementary Material. |
| Missing data | 12c | Missing data | Section 2.5 and Supplementary Material. |
| Loss to follow-up | 12d | Loss to follow-up | Not applicable for the fixed 72-hour retrospective endpoint. |
| Sensitivity analyses | 12e | Sensitivity analyses | Bootstrap internal validation and supplementary analyses. |
| Participants | 13a | Participants | Results section 3.1 and Figure 1. |
| Non-participation | 13b | Non-participation | Eligibility criteria and duplicate exclusion described; add screening/exclusion counts if available. |
| Flow diagram | 13c | Flow diagram | Figure 1. |
| Descriptive data | 14a | Descriptive data | Table 1. |
| Missing data | 14b | Missing data | Table 1 available n and Supplementary Material. |
| Follow-up time | 14c | Follow-up time | Fixed T0-T72 assessment window and secondary hospital outcomes. |
| Outcome data | 15 | Outcome data | Results section 3.1: 102/199 failures. |
| Main results | 16a | Main results | Results sections 3.3-3.5 and Tables 2-4. |
| Category boundaries | 16b | Category boundaries | Most predictors were continuous; transformations are described in Methods and Supplement. |
| Absolute risk | 16c | Absolute risk | Outcome frequency reported as 102/199 (51.3%). |
| Other analyses | 17 | Other analyses | Resource outcomes and exploratory antibiotic findings. |
| Key results | 18 | Key results | Discussion, first paragraph. |
| Limitations | 19 | Limitations | Discussion, final paragraph. |
| Interpretation | 20 | Interpretation | Discussion throughout; causal claims avoided. |
| Generalisability | 21 | Generalisability | Discussion limitations and Conclusion; multicenter validation required. |
| Funding | 22 | Funding | Funding declaration; complete before submission. |
