# Supplementary material for: Dynamic prediction of early antibiotic treatment failure in ischemic stroke-associated pneumonia: development and internal validation of a 48-h landmark model
Source: Ann Med. 2026 Nov 12;58(1):2753847. doi: 10.1080/07853890.2026.2753847 (PMC13761045; doi:10.1080/07853890.2026.2753847)
Supplement: Supplementary file 3 [file IANN_A_2753847_SM5996.docx]

Table S1. Definitions, coding, and preprocessing of model variables.

| **Variable** | **Definition** | **Coding or transformation** | **Assessment time** | **Missing-data and preprocessing rule** |
| --- | --- | --- | --- | --- |
| Primary endpoint | First initial-antibiotic treatment failure occurring after completion of the T48 assessment and by T72. | Binary: 1 = efficacy-driven antibiotic escalation/replacement/addition, failure to achieve clinical stability with documented uncontrolled pneumonia or poor response, or pneumonia-related death; 0 = no failure through T72. | (48,72] h after T0 | Patients had to have an ascertainable T72 outcome. All included patients were event-free at T48. |
| Age | Age at admission. | Age/10; modeled continuously. | Baseline | No missing values. |
| Pre-stroke mRS | Functional status before the index stroke. | Per 1-point increase. | Baseline | One missing value; median imputation within the modeling pipeline. |
| Admission NIHSS | Neurological severity at hospital admission. | NIHSS/5; modeled continuously. | Baseline | One missing value; median imputation within the modeling pipeline. |
| Mechanical ventilation before T0 | Invasive mechanical ventilation initiated before empirical antibiotic treatment. | Binary: 1 = yes; 0 = no. | Before T0 | No missing values. |
| Late AISAP | Stroke-associated pneumonia diagnosed 72-168 h after stroke onset. | Binary: 1 = late AISAP; 0 = diagnosis <72 h. | Baseline | One missing value; median imputation within the modeling pipeline. |
| Diagnosis-to-antibiotic delay | Elapsed time from AISAP diagnosis to first empirical systemic antibiotic dose. | log2(hours + 1). | Baseline | No missing values. |
| Broad-spectrum initial therapy | Initial antipseudomonal beta-lactam/beta-lactamase inhibitor or carbapenem therapy. | Binary: 1 = broad-spectrum; 0 = other initial regimen. | T0 | No missing values; interpreted as an exploratory treatment-selection variable. |
| T0 PLR | Platelet count divided by absolute lymphocyte count. | log2(PLR). | T0 | Calculated only when both components were observed and lymphocyte count was positive; 16 missing values were median-imputed in the modeling pipeline. |
| Delta CRP at T48 | Change in C-reactive protein from T0 to T48. | (CRP at T48 - CRP at T0)/20 mg/L. | T0 to T48 | Calculated only when both measurements were observed; 7 missing values were median-imputed in the T48 model. |
| Delta NLR at T48 | Change in neutrophil-to-lymphocyte ratio from T0 to T48. | (NLR at T48 - NLR at T0)/5; NLR = absolute neutrophils/absolute lymphocytes. | T0 to T48 | Calculated only when all component counts were observed and lymphocyte count was positive; no missing values in the final cohort. |
| Oxygen-support escalation at T48 | Increase in the intensity of oxygen or ventilatory support by the T48 assessment. | Binary: 1 = escalation; 0 = no escalation. | T0 to T48 | No missing values. |

Abbreviations: AISAP, acute ischemic stroke-associated pneumonia; CRP, C-reactive protein; mRS, modified Rankin Scale; NIHSS, National Institutes of Health Stroke Scale; NLR, neutrophil-to-lymphocyte ratio; PLR, platelet-to-lymphocyte ratio; T0, antibiotic initiation; T48, 48-hour landmark; T72, 72-hour assessment. Blank cells were never treated as zero. All predictor transformations were completed before median imputation and standardization.
