# Supplementary material for: Dynamic prediction of early antibiotic treatment failure in ischemic stroke-associated pneumonia: development and internal validation of a 48-h landmark model
Source: Ann Med. 2026 Nov 12;58(1):2753847. doi: 10.1080/07853890.2026.2753847 (PMC13761045; doi:10.1080/07853890.2026.2753847)
Supplement: Supplementary file 4 [file IANN_A_2753847_SM5997.docx]

Table S2. Complete ridge logistic regression specification for the T0 and T48 landmark prediction models.

| **Predictor (transformed scale)** | **Median used for imputation** | **Mean used for standardization** | **SD used for standardization** | **T0 model coefficient beta** | **T48 model coefficient beta** |
| --- | --- | --- | --- | --- | --- |
| Age, per 10 years | 7.000 | 6.935 | 1.117 | 0.031 | 0.140 |
| Pre-stroke mRS, per point | 1.000 | 1.015 | 0.938 | 0.360 | 0.225 |
| Admission NIHSS, per 5 points | 3.000 | 2.928 | 1.104 | 0.272 | 0.121 |
| Mechanical ventilation before T0 | 0.000 | 0.106 | 0.307 | 0.452 | 0.355 |
| Late AISAP | 0.000 | 0.302 | 0.459 | -0.282 | -0.251 |
| Diagnosis-to-antibiotic delay, log2(hours + 1) | 0.874 | 1.828 | 1.806 | -0.255 | -0.080 |
| Broad-spectrum initial therapy | 0.000 | 0.422 | 0.494 | -0.578 | -0.318 |
| T0 PLR, log2 | 7.763 | 7.854 | 0.802 | 0.332 | 0.771 |
| Delta CRP at T48, per 20 mg/L | 0.650 | 1.131 | 3.575 | Not included | 1.973 |
| Delta NLR at T48, per 5 units | -0.347 | -0.516 | 1.824 | Not included | 0.977 |
| Oxygen-support escalation at T48 | 0.000 | 0.176 | 0.381 | Not included | 0.492 |
| Intercept | Not applicable | Not applicable | Not applicable | 0.075 | 0.358 |

Model calculation. For each predictor, first apply the transformation shown in Supplementary Table S1. If a value is missing, replace it with the listed median. Standardize the resulting value as z = (x - mean)/SD. The linear predictor is eta = intercept + sum(beta x z), and the predicted probability is P = 1/[1 + exp(-eta)].
Interpretation. The coefficients are penalized ridge-logistic coefficients used to generate predicted probabilities; they are not the conventional logistic-regression coefficients underlying the adjusted odds ratios in the main manuscript.
Model stages. The T0 model uses baseline variables only. The T48 landmark model retains all T0 predictors and adds Delta CRP, Delta NLR, and oxygen-support escalation measured by T48.
Internal validation. Model performance was internally validated with 500 bootstrap resamples. External validation and local recalibration are required before clinical implementation.
