# Supplementary material for: Dynamic prediction of early antibiotic treatment failure in ischemic stroke-associated pneumonia: development and internal validation of a 48-h landmark model
Source: Ann Med. 2026 Nov 12;58(1):2753847. doi: 10.1080/07853890.2026.2753847 (PMC13761045; doi:10.1080/07853890.2026.2753847)
Supplement: Supplementary file 5 [file IANN_A_2753847_SM6001.docx]

Table S3. Nonexclusive composite failure components and available microbiology (n=199)

| **Outcome or measurement** | **n (%) / value** | **Notes** |
| --- | --- | --- |
| First composite antibiotic-treatment failure | 102 (51.3%) | Of the entire cohort (n=199) |
| Efficacy-driven antibiotic escalation / replacement / addition | 70 (35.2%) | 68.6% of 102 composite failures; nonexclusive |
| Persistent instability at T72 with documented poor response | 99 (49.7%) | 97.1% of 102 failures; nonexclusive |
| Both antibiotic modification and persistent instability | 67 (33.7%) | Counted once in composite |
| Only antibiotic modification | 3 (1.5%) | Removed by instability-only sensitivity outcome |
| Only instability | 32 (16.1%) | No recorded efficacy-driven antibiotic change |
| Pneumonia-related death by the nominal T72 endpoint | 0 (0.0%) |  |
| Culture positive / negative / unavailable | 33 / 121 / 45 |  |
| Multidrug resistance yes / no | 13 / 20 |  |
