# Supplementary material for: Dynamic prediction of early antibiotic treatment failure in ischemic stroke-associated pneumonia: development and internal validation of a 48-h landmark model
Source: Ann Med. 2026 Nov 12;58(1):2753847. doi: 10.1080/07853890.2026.2753847 (PMC13761045; doi:10.1080/07853890.2026.2753847)
Supplement: Supplementary file 6 [file IANN_A_2753847_SM6002.docx]

Table S5. Original-scale continuous predictor distributions and functional-form assessments

| **Original-scale variable** | **Observed / missing** | **Entire cohort median (IQR)** | **Success (n=97) median (IQR)** | **Failure (n=102) median (IQR)** | **Observed range** |
| --- | --- | --- | --- | --- | --- |
| Age, years | 199 / 0 | 70 (63–76.50) | 70 (62–77) | 71 (65–75.75) | 34–98 |
| Pre-stroke mRS, points | 198 / 1 | 1 (0–2) | 1 (0–1) | 1 (0–2) | 0–4 |
| Admission NIHSS, points | 198 / 1 | 15 (10.25–18) | 14.50 (9–17) | 16 (13–19) | 3–29 |
| Diagnosis-to-antibiotic delay, hours | 199 / 0 | 0.83 (0.50–10) | 1 (0.50–13) | 0.67 (0.50–6.75) | 0–118 |
| Baseline PLR | 183 / 16 | 217.23 (153.14–332.64) | 200.68 (139.67–297.89) | 238.61 (176.98–366.25) | 35.59–1587.30 |
| CRP at T0, mg/L | 195 / 4 | 47 (21–83) | 65 (27–102) | 35 (14.75–72.85) | 0.64–240 |
| CRP at T48, mg/L | 195 / 4 | 69 (34.45–127.50) | 38 (20.50–64.50) | 118 (73–172.75) | 0.50–285 |
| ΔCRP (T48 − T0), mg/L | 192 / 7 | 13 (-25.18–65.65) | -20.50 (-49.38–0.95) | 63.05 (20.25–108.18) | -144–235 |
| NLR at T0 | 199 / 0 | 9.43 (6.15–14.85) | 8.69 (5.87–12.70) | 11.60 (6.50–16.18) | 1.53–58.90 |
| NLR at T48 | 199 / 0 | 7.59 (4.96–11.56) | 5.89 (4.01–8.09) | 9.59 (6.97–15.00) | 0.74–54.75 |
| ΔNLR (T48 − T0) | 199 / 0 | -1.74 (-5.24–0.49) | -2.56 (-5.41–-0.78) | -0.08 (-4.74–2.74) | -38.82–48.17 |

Panel A. Observed clinical measurements before transformation or imputation (n = 199).

Panel B. Exploratory adjusted tests of nonlinearity in modeled continuous predictors.

| **Predictor** | **Primary modeling form** | **T0 nonlinearity P** | **T48 nonlinearity P** |
| --- | --- | --- | --- |
| Age | age/10 | 0.920 | 0.076 |
| Admission NIHSS | NIHSS/5 | 0.956 | 0.965 |
| Diagnosis-to-antibiotic delay | log₂(hours + 1) | 0.653 | 0.941 |
| Baseline PLR | log₂(PLR) | 0.521 | 0.086 |
| ΔCRP | ΔCRP/20 mg/L | — | 0.050 |
| ΔNLR | ΔNLR/5 | — | 0.616 |
| Pre-stroke mRS (ordinal sensitivity) | Linear per-point vs categorical | 0.498 | 0.662 |
