# Supplementary material for: Dynamic prediction of early antibiotic treatment failure in ischemic stroke-associated pneumonia: development and internal validation of a 48-h landmark model
Source: Ann Med. 2026 Nov 12;58(1):2753847. doi: 10.1080/07853890.2026.2753847 (PMC13761045; doi:10.1080/07853890.2026.2753847)
Supplement: Supplementary file 7 [file IANN_A_2753847_SM7632.docx]

**SUPPLEMENTARY FIGURE LEGENDS**

Supplementary Figure S1. Adjusted odds ratios for the 48-hour landmark model. Error bars indicate 95% confidence intervals.

Supplementary Figure S2. C-reactive protein trajectory by treatment outcome. Lines show medians and shaded regions show interquartile ranges.

Supplementary Figure S3. Neutrophil-to-lymphocyte ratio trajectory by treatment outcome. Lines show medians and shaded regions show interquartile ranges.

Supplementary Figure S4. Platelet-to-lymphocyte ratio trajectory by treatment outcome. Lines show medians and shaded regions show interquartile ranges.

Supplementary Figure S5. Hospital length of stay according to treatment outcome.

Supplementary Figure S6. Bootstrap optimism-corrected discrimination across full and oxygen-excluded T48 models for the original composite endpoint and an alternative T72-instability endpoint (500 resamples). Values are descriptive and are not tests of between-model differences.
